# Prevalence, characteristics, and impact on health outcomes of frailty in elderly outpatients with diabetes: A cross-sectional study

**DOI:** 10.1097/MD.0000000000036187

**Published:** 2023-11-24

**Authors:** Qinqin Wang, Juan Wang, Guizhi Dai

**Affiliations:** a Outpatient Department, Deyang People’s Hospital, Deyang, China.

**Keywords:** diabetes, elderly, frailty, health outcome, outpatient

## Abstract

The aims of this study were to determine the prevalence of frailty and its relationship with health outcomes in elderly outpatients attending a Diabetes Specialist Clinic. This study was a cross-sectional study. A total of 168 elderly patients (aged 65 years and above) attending the Diabetes Specialist Clinic of a Three-A hospital of Sichuan province were recruited from January 2021 to February 2021, and follow-up was conducted 1 year after day of screening. Baseline characteristics of patients were collected and frail status were assessed at recruitment. The longitudinal outcomes included hospitalization, fall, mortality, emergency visit, and clinic visit. The presence of frailty was determined by the 5-item FRAIL scale, which ranges from 0 to 5 and are categorized as frail (3–5), prefrail (1–2), and robust (0). A phone questionnaire was carried out to obtain health outcomes. Logistic regression analyses was used to evaluate adverse health outcomes at 1 year follow-up. Of the 168 outpatients, 28.0% was robust, 49.4% was prefrail, and 22.6% was frail. Frailty (both prefrail and frail status) was more prevalent in those patients, which were 75 years old and above (57.0%; *P* < .001), insulin dependent (45.6%; *P* = .008), and those had diabetic complications (43.8%; *P* = .005), previous admission (68.6%; *P* = .016), and co-morbidities (36.4%; *P* = .001). In the following year after recruitment, 19.1% of robust patients were hospitalized, while the proportion was 45.8% for prefrail patients and 65.8% for frail patients. Prefrail (OR [odds ratio] = 2.35, 95% confidence interval (CI) 1.63–2.88; *P* = .028) and frail (OR = 4.63, 95% CI 2.52–5.81; *P* = .005) patients were more likely to be hospitalized. Frail (OR = 3.37, 95% CI 2.68–4.04; *P* < .001) patients were more inclined to fall while prefrail patients (OR = 1.03, 95% CI 0.82–1.56; *P* = .371) were not. Moreover, prefrail (OR = 3.37, 95% CI 2.31–5.72; *P* = .017) and frail (OR = 4.29, 95% CI 3.16–5.54; *P* = .006) patients were more likely to return to the clinic. There is a high incidence of frailty among elderly patients attending a Diabetes Specialist Clinic. Frailty is a predictor of hospitalization, fall, and clinic visits within 1 year.

## 1. Introduction

The incidence of diabetes in the elderly population is as high as 25% although there are regional and ethnic differences. The trend of global prevalence is increasing with an estimated double number of cases of diabetes among older adults in the next 2 decades.^[1]^ The aging process of China’s population is obvious, and the number of elderly patients with diabetes have increased a lot. The management of such patients is a challenge.

Frailty, which means a decline in functioning across multiple physiological systems and an increased vulnerability to stressors, is common in older adults, especially those with diabetes.^[2,3]^ Frailty can reflect the function and overall situation of elderly patients, and has been used to predict adverse outcomes for older patients with diabetes.^[4–6]^ Effective identification and diagnosis of frailty and appropriate interventions can reduce the readmission rate, thereby reducing the consumption of medical resources and reducing medical insurance expenditures.^[7,8]^ However, the assessments of frailty after admission and subsequent interventions are actually a little backward, or can be called “tertiary prevention,” while screening and interventions in the diabetes specialist clinic can achieve the effect of “secondary prevention.” Due to the large population base in China and the lack of community medical services, it is of great significance to identify and manage the frailty in outpatients, especially for those with diabetes. Frailty status and its association with adverse health outcomes in elderly outpatients with diabetes should be clarified before effective interventions. Studies show that elderly people with diabetes have a high incidence of frailty, which means a greater economic burden.^[9]^

Previous studies have shown that frailty is an independent risk factor for adverse health outcomes such as readmission.^[10]^ Studies have shown that the incidence of frailty in elderly outpatients with diabetes aged 50 to 90 years is high and can be used as a predictor of admission risk.^[11]^ There were no relevant studies in China, and previous studies mainly focus on the relationship between frailty and death, admission risk, loss of ADL, and emergency visit risk. Moreover, there was no study on frailty predicting the risk of outpatient revisit and fall in elderly patients with diabetes. The primary aim of this study was to use the FRAIL scale to determine the prevalence of frailty among older outpatients with diabetes in China and to explore the relationship between frailty and adverse health outcomes such as hospitalization, fall, emergency visit, clinic visit.

## 2. Method

### 2.1. Study design

This study used a cross-sectional research design to discern prevalence and characteristics of frailty in elderly outpatients with diabetes. And the aim of this study was to determine the relationship between frailty and health outcomes in elderly outpatients attending a Diabetes Specialist Clinic.

### 2.2. Setting and population

A cross-sectional study was conducted in the Diabetes Specialist Clinic of People’s Hospital of Deyang City. The inclusion criteria were as follows: ① The period was from January 1, 2021 to February 28, 2021; ② In the diabetes specialist clinic; ③ Elderly patients aged 65 years and above; ④ Type I or type II diabetes. Patients with the following conditions were excluded: ① patients less than 65 years old; ② Non-diabetic patients. After screening, a total of 180 patients were eligible, and 168 patients agreed to participate in the survey (participation rate was 93.3%). All 168 patients completed the survey and follow up. This retrospective study was approved by the ethics committee of Deyang Peoples’ Hospital, an Affiliated Hospital of Chengdu University of TCM and informed consent was obtained from all the participants. This study did not receive any funding support.

### 2.3. Instruments

#### 2.3.1. FRAIL scale.

FRAIL scale, which included the following 5 components: Fatigue, Resistance, Ambulation, Illness, and Loss of weight was used to assess frailty of all participants at the recruitment. Each item is allocated 1 point then FRAIL scores represent frail (3–5), prefrail (1–2), robust (0). Each component was conducted by asking patients 1 question. Fatigue is measured by asking how much of the time participants feel tired in the past 2 weeks; responses of “all” or “most of the time” are scored 1 point. Resistance is measured by asking participants if they have difficulty walking up 10 steps without resting, and ambulation is measured by asking participants if they have difficulty walking 1 block; yes responses are each scored 1 point. Illnesses is measured by asking participants if a physician has ever told them they had the following illnesses: hypertension, cancer (other than minor skin cancer), diabetes, chronic lung disease, heart attack, congestive heart failure, angina, asthma, arthritis, stroke, kidney disease; responses are scored 1 point if participants report more than 5 illnesses. Loss of weight is measured by asking participants how much they weighed on the day of screening and how much they weighed 6 months ago; loss of weight of 5% or more over is scored 1 point.

#### 2.3.2. Demographic variables.

Demographic information included age, sex, BMI, and smoking history. Age was classified into 2 categories: 65 to 74 years and ≥75years. Smoking history was classified into 2 categories: current smoking or not.

#### 2.3.3. Health-related variables.

Clinical data consisted of duration of diabetes, current insulin dependent, diabetic complications, HbA1c, previous falls, previous hospitalization, and comorbidities.

#### 2.3.4. Outcome variable.

One year after enrollment, all patients (or family members) were followed by telephone for primarily adverse health outcomes, including readmission, falls, clinic visit, emergency visit, and death. All outcome events were dichotomous variables.

### 2.4. Sample size and data collection

According to previous reports, a rough estimation method of sample size was used: 10 times the number of study variables was used to calculate the sample size. There were 13 variables in the study, hence, the sample size of this study was 130 patients. Considering a loss rate of 20%, the minimum sample size of the study was 163 patients. We used Microsoft Excel 2010 software to collect data.

### 2.5. Statistical analysis

All data were analyzed using IBM SPSS Statistics, (version 23, Chicago). Counting data are expressed as frequencies or percentages, and the chi-square test was used for comparison between groups. The measured data were first tested for normality. If a normal distribution was satisfied, the mean ± standard deviation was used to represent the data and *t* test was performed between groups, while if not, the rank sum test was used. Logistic regression analyses was used to evaluate adverse health outcomes at 1 year follow-up. Adjusted odds ratios (ORs) and 95% confidence intervals (CIs) are reported for logistic regression analyses. *P* < .05 indicates a statistically significant difference.

## 3. Results

A total of 180 people were eligible for the study. Except for 12 people who did not want to participate in the survey, 168 people successfully completed the study, with a follow-up rate of 100 percent. Among them, 87 (51.8%) were female, 76 (45.2%) were aged 75 years and over, and 105 (62.5) had previous hospitalization experience. As shown in Table 1. There were 38 frail patients (22.6%) and 83 prefrail patients (49.4%). There was no significant difference in frailty in terms of gender, BMI, smoking, duration of diabetes, glycated hemoglobin level, and past history of falls. However, there were differences in age, insulin dependence, diabetic complications, previous hospitalization history, and comorbidities. Frailty (both prefrail and frail status) was more prevalent in those patients, which were 75 years old and above (57.0%; *P* < .001), insulin dependent (45.6%; *P* = .008), and those had diabetic complications (43.8%; *P* = .005), previous admission (68.6%; *P* = .016), and co-morbidities (36.4%; *P* = .001).

**Table 1:** Characteristics of patients by frailty.

Characteristics	Total	Frail			*P*-value
		Robust	Prefrail	Frail	
Sex, n (%)					.183
Male	81 (48.2)	28 (59.6)	36 (43.4)	17 (44.7)	
Female	87 (51.8)	19 (40.4)	47 (56.6)	21 (55.3)	
Age (yr), n (%)					<.001
65–74	92 (54.8)	40 (85.1)	42 (50.6)	10 (26.3)	
≥75	76 (45.2)	7 (14.9)	41 (49.4)	28 (73.7)	
BMI (kg/m^2^)	23.18 ± 2.12	22.95 ± 2.67	23.24 ± 3.02	23.32 ± 2.53	.702
Smoking, n (%)					.603
Yes	43 (25.6)	11 (23.4)	24 (28.9)	8 (21.1)	
No	125 (74.4)	36 (76.6)	59 (71.1)	30 (78.9)	
Insulin dependent, n (%)					.008
Yes	68 (40.5)	13 (27.7)	32 (38.6)	23 (60.5)	
No	100 (59.5)	34 (72.3)	51 (61.4)	15 (39.5)	
Diabetic complication, n (%)					0.005
Yes	64 (36.3)	8 (17.0)	35 (42.2)	18 (47.4)	
No	104 (63.7)	39 (83.0)	48 (57.8)	20 (52.6)	
Duration of diabetes (yr), n (%)					.308
<10	96 (57.1)	30 (63.8)	48 (57.8)	18 (47.4)	
>10	72 (42.9)	17 (36.2)	35 (42.2)	20 (52.6)	
HbA1c(%)	9.02 (8.48–9.67)	8.28 (7.54–8.95)	9.12 (8.67–9.90)	9.43 (8.56–10.06)	.512
Previous falls, n (%)					.407
Yes	45 (26.8)	10 (21.3)	22 (26.5)	13 (34.2)	
No	123 (73.2)	37 (78.7)	61 (73.5)	25 (65.8)	
Previous admission, n (%)					.016
Yes	105 (62.5)	22 (46.8)	54 (65.1)	29 (76.3)	
No	63 (37.5)	25 (53.2)	29 (34.9)	9 (23.7)	
Co-morbidity, n (%)					.001
Yes	52 (31.0)	8 (17.0)	23 (27.7)	21 (55.3)	
No	116 (69.0)	39 (83.0)	60 (72.3)	17 (44.7)	

**Figure 1.**
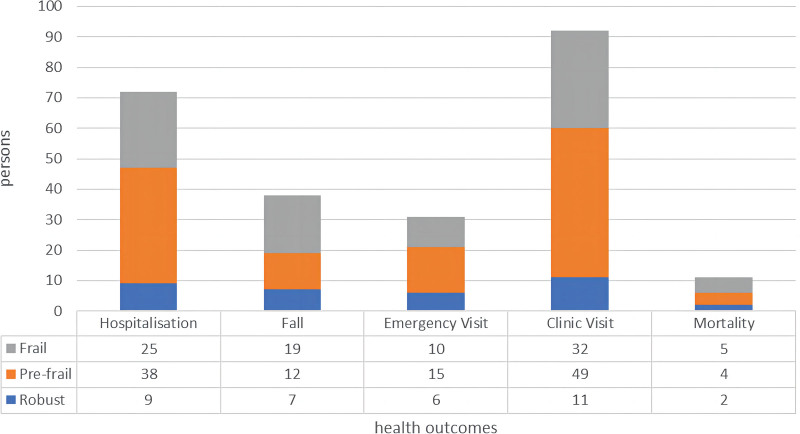
Health outcomes in varying health status.

**Figure 2.**
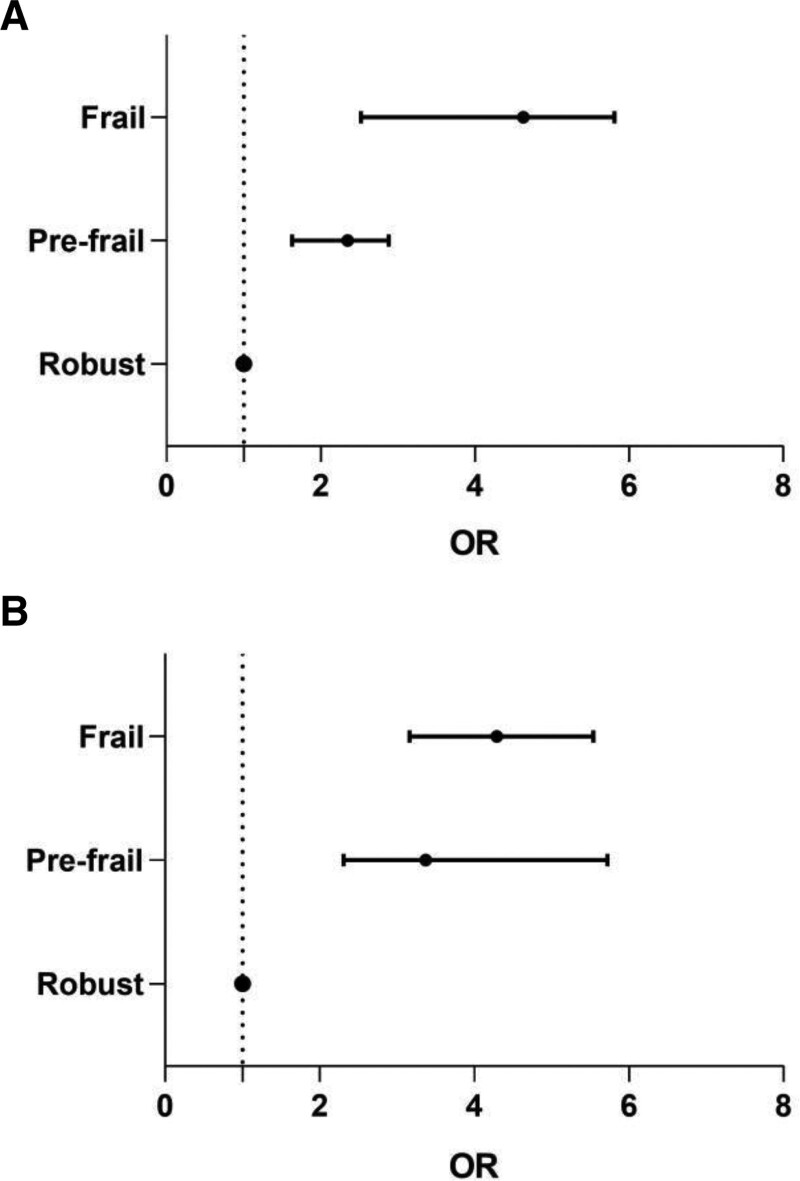
(A) Hospitalization risk by varying frailty. (B) Clinic visit risk by varying frailty.

In terms of health outcomes (Table 2, Fig. 1), within 1 year, 72 were re-hospitalized, 8 died, 38 fell, 31 visited the emergency department at least once, and 92 returned to the outpatient clinic at least once. There were statistically significant differences in readmission, falls and outpatient visits. Adjusted for sex, age, BMI, Insulin dependent, HbA1c, and Co-morbidity, the Prefrail (OR = 2.35, 95% CI 1.63–2.88; *P* = .028) and frail (OR = 4.63, 95% CI 2.52–5.81; *P* = .005) patients were more likely to be hospitalized. Frail (OR = 3.37, 95% CI 2.68–4.04; *P* < .001) patients were more inclined to fall while prefrail patients (OR = 1.03, 95% CI 0.82–1.56; *P* = .371) were not. Moreover, prefrail (OR = 3.37, 95% CI 2.31–5.72; *P* = .017) and frail (OR = 4.29, 95% CI 3.16–5.54; *P* = .006) patients were more likely to return to the clinic as shown in Table 3 and Figure 2.

**Table 2:** Health outcomes at 1 year follow-up.

	Hospitalisation		Fall		Emergency visit		Clinic visit	
	Yes	No	Yes	No	Yes	No	Yes	No
Robust, n (%)	9 (12.5)	38 (39.6)	7 (18.4)	40 (30.8)	6 (19.3)	41 (30.0)	11 (11.9)	36 (47.4)
Prefrail, n (%)	38 (52.8)	45 (46.9)	12 (31.6)	71 (54.6)	15 (48.4)	68 (49.6)	49 (53.3)	34 (44.7)
Frail, n (%)	25 (34.7)	13 (13.5)	19 (50.0)	19 (14.6)	10 (32.3)	28 (20.4)	32 (34.8)	6 (7.9)
Total, n (%)	72 (100)	96 (100)	38 (100)	130 (100)	31 (100)	137 (100)	92 (100)	76 (100)
*P* value	<.001		<.001		.275		<.001	

**Table 3:** Rates, ORs, and 95% CIs for outcomes during the past year.

Outcomes	Robust (N = 47)	Prefrail (N = 83)		Frail (N = 38)	
		OR (95% CI)	*P* value	OR (95% CI)	*P* value
Hospitalization					
Rate (%)	19.1	38.7		65.8	
Adjusted OR*	Reference	2.35 (1.63–2.88)	.028	4.63 (2.52–5.81)	.005
Fall					
Rate (%)	14.9	14.5		50	
Adjusted OR†	Reference	1.03 (0.82–1.56)	.371	3.37 (2.68–4.04)	<.001
Clinic visit					
Rate (%)	23.4	59		84	
Adjusted OR‡	Reference	3.37 (2.31–5.72)	.017	4.29 (3.16–5.54)	.006

CI = confidence interval, OR = odds ratio.

* Adjusted for sex, age, BMI, Insulin dependent, HbA1c, and co-morbidity.

† Adjusted for sex, age, previous falls, and co-morbidity.

‡ Adjusted for age, smoking, Insulin dependent, HbA1c, diabetic complication, previous falls, and co-morbidity.

## 4. Discussion

Elderly diabetic patients with or without organ dysfunction have a high incidence of frailty. Frailty has been proved strongly associated with adverse health outcomes.^[12]^ Therefore, older people with diabetes and frailty are at higher risk for adverse health outcomes.^[13,14]^ This study examined the cross-sectional association between frailty and risk of adverse health outcomes among elderly diabetic outpatients. The results showed that frail elderly diabetic outpatients were at an increased risk for hospitalization, fall, and clinic visit. A review of the literatures showed that there were no previous studies on this point.

In this study, the prevalence of frailty among older outpatients attending a Diabetes Specialist Clinic was 72.0%, among which the proportion of frail patients was 22.6%, while it was 49.4% for prefrail patients. The result is consistent with previous studies.^[15–17]^ However, a pilot study had reported that the incidence of frailty in elderly in patients with diabetes in China was 52.8%.^[10]^ The reason for this difference may be that the participants in present study were over 65 years old, while those were over 60 years old in the mentioned research and previous studies had shown that increasing age itself increases the risk of frailty.^[18]^ Another study showed that the prevalence of frailty among community-dwelling older adults with diabetes assessed by FRAIL Scale was 44.7%,^[9]^ which indicates that the incidence of frailty in elderly outpatients with diabetes is higher than that in community-dwelling elderly patients with diabetes. In present study, age, insulin dependence, diabetic complications, previous hospitalization history, and comorbidities are related to frailty in elderly outpatients attending a Diabetes Specialist Clinic. In general, frailty is a symptom, which means a decline in functioning across multiple physiological systems and an increased vulnerability to stressors.^[19]^ Some studies have shown that insulin resistance is also one of the reasons for the high incidence of frailty in diabetic patients.^[20]^ The subjects of present study were all elderly patients, most of whom were characterized by type 2 diabetes, so we didn’t product more about insulin resistance.

The results showed that frail patients had a significantly higher risk of hospitalization within 1 year after recruitment, with a OR 2.35 (95% CI 1.63–2.88) for prefrail patients and 4.63 (95% CI 2.52–5.81) for frail patients compared to robust patients. And frail patients were more inclined to fall with a OR 3.37 (95% CI 2.68–4.04) compared to robust patients in the following year. Moreover, frail and prefrail patients were more likely to return to the clinic in comparison with robust elderly outpatients with diabetes. The OR was 3.37 (95% CI 2.31–5.72) for prefrail patients and 4.29 for frail patients (95% CI 3.16–5.54). Therefore, frailty assessed by FRAIL Scale can be used as an independent risk factor for hospitalization and frequent clinic visit in elderly outpatients with diabetes.

There are a large number of diabetic outpatients in Chinese hospitals, and the number of elderly patients with diabetes is gradually increasing. Reducing the hospitalization rate and outpatient volume of elderly diabetic patients can bring huge social and economic benefits. Previous studies on frailty in elderly patients with diabetes mostly focused on community residents and inpatients, and there was a lack of relevant studies on predicting the adverse health outcomes of outpatients. Our study shows that frailty assessed by FRAIL Scale can effectively predict the risk of hospitalization, fall, and clinic revisit in elderly outpatients with diabetes, which provides a theoretical basis for proposing effective interventions at the outpatient for health care workers.

## 5. Limitations

There are some shortcomings in our study. It was a single-center cross-sectional study, so the characteristics of the participants such as race and region may lead to bias. More research is worth doing.

## 6. Conclusion

There is a high incidence of frailty among elderly patients attending a Diabetes Specialist Clinic. Frailty is a predictor of hospitalization, fall, and clinic visits within 1 year.

## Acknowledgments

We thank Liu Die for helping with data collection, Chen Yanyu, and Wu Linxia for helping with the follow up work, and Zou Huixiang for quality assurance of the research. Although all the members above did not participate in the writing of the article, they did a lot of help.

## Author contributions

**Conceptualization:** Qinqin Wang.

**Data curation:** Juan Wang.

**Formal analysis:** Qinqin Wang.

**Investigation:** Qinqin Wang, Juan Wang, Guizhi Dai.

**Methodology:** Juan Wang.

**Project administration:** Guizhi Dai.

**Resources:** Guizhi Dai.

**Software:** Qinqin Wang.

**Validation:** Guizhi Dai.

**Visualization:** Guizhi Dai.

**Writing – original draft:** Qinqin Wang.

**Writing – review & editing:** Qinqin Wang.
